# Anatomical Confirmation of Computed Tomography-Based Diagnosis of the Atherosclerosis Discovered in 17^th^ Century Korean Mummy

**DOI:** 10.1371/journal.pone.0119474

**Published:** 2015-03-27

**Authors:** Myeung Ju Kim, Yi-Suk Kim, Chang Seok Oh, Jai-Hyang Go, In Sun Lee, Won-Kyu Park, Seok-Min Cho, Soon-Kwan Kim, Dong Hoon Shin

**Affiliations:** 1 Department of Anatomy, Dankook University College of Medicine, Cheonan, Korea; 2 Department of Anatomy, Ewha Womans University School of Medicine, Seoul, Korea; 3 Department of Anatomy, Seoul National University College of Medicine, Seoul, Korea; 4 Institute of Forensic Science, Seoul National University College of Medicine, Seoul, Korea; 5 Department of Pathology, Dankook University College of Medicine, Cheonan, Korea; 6 Department of Radiology, Seoul National University Hospital, Bundang, Korea; 7 Department of Wood and Paper Science, Chungbuk National University, Cheongju, Korea; 8 Gaya National Research Institute of Cultural Heritage, Changwon, Korea; 9 National Research Institute of Cultural Heritage, Cultural Heritage Science Center, Daejeon, Korea; Massachusetts General Hospital and Harvard Medical School, UNITED STATES

## Abstract

In the present study on a newly discovered 17^th^ century Korean mummy, computed tomography (CT) revealed multiple aortic calcifications within the aortic wall that were indicative of ancient atherosclerosis. The CT-based findings were confirmed by our subsequent *post-factum* dissection, which exhibited possible signs of the disease including ulcerated plaques, ruptured hemorrhages, and intimal thickening where the necrotic core was covered by the fibrous cap. These findings are strong indicators that the mummy suffered from aortic atherosclerosis during her lifetime. The present study is a good example of how CT images of vascular calcifications can be a useful diagnostic tool in forming at least preliminary diagnoses of ancient atherosclerosis.

## Introduction

Atherosclerosis is characterized by intimal lesions called atheromatous plaques, which protrude into the vascular lumens where they can cause obstruction and sometimes, further complications thereby. Although these lesions begin as benign fatty streaks that do not interfere with blood flow, they eventually progress to atheroma, fibroatheroma, and other lesions. The prevalence and severity of the disease among specific individuals and groups are known to be closely related to a number of constitutional and acquired factors. Generally, the incidence of atherosclerosis is significantly high in developed countries [1].

Interestingly, atherosclerosis is also encountered in paleopathological studies on ancient or medieval mummies. The first finding of atherosclerosis, confirmed by careful review of the morphological evidence, was made in several thousand-year-old Egyptian mummies at the beginning of the 20th century [2]. Since that time, atherosclerosis detection in mummies has both improved and accelerated, thanks to rapid developments in various scientific techniques.

Computed tomography (CT)-based paleopathological studies have made remarkable contributions to the construction of a comprehensive understanding of atherosclerosis in human history. Briefly, Allam et al. [3–5] performed whole-body CT scanning of ancient Egyptian mummies, examining calcifications within vascular walls for possible signs of atherosclerosis. Thompson et al. [6] used CT imaging to identify suggestive pathognomonic signs of ancient atherosclerosis, namely calcifications localized on vascular walls or along the expected course of arteries Abdelfattah et al. [7] investigated the case of an atherosclerotic Egyptian woman estimated to have lived about 3,000 years ago, discovering calcified arterial atherosclerotic plaques. Most recently, Piombino-Mascali et al. [8] used Thompson et al.’s protocol [6] to isolate vascular calcifications indicative of atherosclerosis in 18^th^ to 19^th^ century Lithuanian mummies. CT diagnostics now becomes a commonly employed tool for the detection of atherosclerosis-suggestive signs (*i*.*e*. calcifications) in mummies, and is highly valued for its utility in limiting tissue damage.

However, we must also acknowledge some researchers’ concerns about the application of CT analysis to the diagnosis of ancient atherosclerosis. They have pointed out that vascular calcification has causal pathological conditions other than atherosclerosis. They have recommended additional histological studies for differentiation of such findings [9, 10]. In a sense, the criticism of the technical limitations of radiological diagnosis for mummy atherosclerosis deserves consideration. It should be conceded that clinical radiologists’ CT-based interpretations have been supplemented and improved by means of the continuous feedback of *post-factum* autopsy data [11].

Unfortunately, however, obtaining permission for mummy dissection, whether from civic-institutional authorities or familial descendants, usually is far from easy in most countries [11]. Correct interpretation of calcifications on mummy CT images thus far has been hampered by the lack of sufficient available and relevant dissection data. Accumulation of as much *post-factum* dissection data as possible becomes essential to definite diagnosis of ancient atherosclerosis in mummy studies.

Recently, we had the unique opportunity to examine a quite well-preserved 17^th^ century Korean mummy. On initial CT radiography, we found multiple calcifications within the aortic wall, on which basis we suspected ancient atherosclerosis in this case. We then needed to subject the calcifications on CT images to the kind of close scrutiny possible only by means of dissection, by which we were able to confirm the preliminary diagnosis of atherosclerosis. And fortunately, in regard to this case, we were able to utilize the precision tool of dissection, pursuing the purpose of partially fulfilling the goal of improving radiological readings on the vascular calcifications in mummies. The current study can provide invaluable data on the diagnostic value of CT analysis in studies on ancient atherosclerosis.

## Materials and Methods

### The Mummy

**Fig 1:**
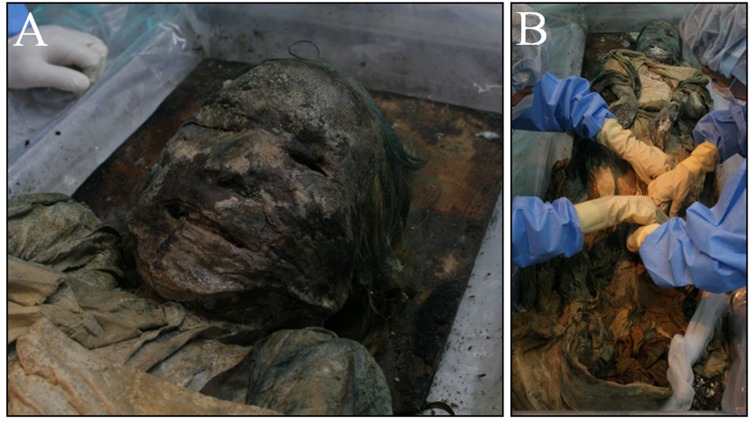
Female mummy discovered in a Joseon tomb. (A) A female mummy examined in this study. (B) Textile specialists removed clothing wrapped around the mummy.

In April 2010, a female mummy (nick-named the Mungyeong mummy; repository number #278 in Joseon Dynasty Human Sample Collection of Seoul National University College of Medicine, Seoul, South Korea) was discovered in a Joseon tomb that had been interred in what is now Mungyeong County, South Korea (Fig. 1A). A tree-ring test [12] dated the wood of the outermost coffin to be about 1647 CE. Considering the period needed for wood drying and processing, the coffin was determined to have been constructed most likely in the 1650s. All necessary permits were issued by Archaeological Policy Division, Cultural Heritage Administration of Korea for the described study (#5383, April 20. 2010; #5651, April 24. 2010), which complied with all relevant regulations. Under the auspices of the Gyeongju Research Institute of Cultural Heritage, the mummy was moved to our lab for further anthropological study. Our anatomical, histological and radiological investigations were authorized by the Institutional Review Board (IRB) of Seoul National University Hospital (H-1108-049-120). This study was conducted in accordance with the Vermillion Accord on Human Remains, World Archaeological Congress (South Dakota, 1989).

### Anthropological Examination

Textile specialists removed the clothing that had been wrapped around the body (Fig. 1B). The subsequent anthropological examination proceeded as follows. Briefly, the sex determination was made on the basis of the morphology of the pelvic bone. To determine the pelvic dimorphism, we examined the greater sciatic notch, the pre-auricular sulcus, the ischiopubic ramus, the subpubic angle, the subpubic concavity, and the ventral arc [13, 14]. The mummy’s age was estimated correlatively to the degeneration of the auricular and pubic symphyseal surfaces of the hip bone [15, 16], according to the categories of young (20–35 years), middle (35–50 years), and old-aged adult (over 50 years). Supplementarily, Lamendin’s age-estimation method [17] was also applied to the mummy’s single-root tooth (right maxillary premolar). With the tooth placed on a light board, the periodontosis height, transparency height, and root height were measured with a vernier caliper. Using a simple formula (Age in years = 0.18 × P + 0.42 × T + 25.53; P = periodontosis height/root height × 100; T = transparency height/root height × 100), the age at death was estimated. An anthropometric examination following Martin’s method [18] also was performed.

### Computed Tomography

CT scanning was performed with a 64 MDCT scanner (VCT; GE Healthcare, Little Chalfont, United Kingdom) at Seoul National University Hospital using the helical technique (120kVP) to acquire a spiral volume from head to toe. All of the data were reconstructed into axial images (thickness: 1.25 mm; interval: 1.25 mm) that were then transferred to a workstation (Advantage Windows Workstation 4.3; GE Healthcare, Little Chalfont, United Kingdom) preparatory to post-processing, by which coronal and sagittal multi-planar reformation and volume-rendering images were obtained.

### *Post-factum* Dissection

We dissected the mummy in the Department of Anatomy, Seoul National University College of Medicine, South Korea. The first incision was made along the lower borders of the xyphoid process and the 12^th^ ribs, and the second, from the lower tip of the xyphoid process and running alongside the *linea alba*. The skin was then pulled apart to expose the internal organs in the abdominal cavity. The thoracic cavity was then opened by cutting the costal cartilages of the ribs and incising the costal or sternal origins of the diaphragmatic muscles. The sternum was then bent back to reveal the organs within the chest cavity.

The calcifications noted on the CT radiological images were counter-checked by the dissection findings, specifically by examining the ascending and descending aorta and aortic arch. The vessel walls were cut and their insides exposed to view. Cross-sections of the atheroma were also made, for comparison with the corresponding calcifications on the CT images. Finally, we searched for signs of coronary atherosclerosis in our subject. The anterior side of the heart was dissected, cross-sectioning the left anterior descending (LAD) artery.

A portion of LAD artery was sampled for further histological study. The sample was rehydrated in Ruffer’s solution (distilled water: absolute ethanol: 5% sodium carbonate, 5: 3: 2) for 72 h [19]; after fixation in 4% paraformaldehyde in phosphate-buffered saline (pH 7.2), it was washed with phosphate-buffered saline (pH 7.2), embedded in optimal cutting temperature compound (Sakura; Torrance, CA, USA), and cut into 5- or 12-μm sections on a cryostat (Leica; Nussloch, Germany) [20]. Hematoxylin/eosin (H&E) and Masson’s trichrome staining were performed on the obtained slides, as described in previous studies [21, 22].

## Results

The mummy’s sex (female) was determined initially according to the external genitalia. This conclusion was later confirmed synthetically with reference to the hip-bone morphological findings: widened sciatic notch; existence of pre-auricular sulcus; and sharpness of ischiopubic ramus (S1 Table). As for the age at death, the mummy was deemed to have been middle-aged (35–50 years), based on the degeneration of the auricular and pubic symphyseal surfaces of the hip bone. The age estimated by supplementary Lamendin method [17], which is regarded as the best age-estimation modality for the middle-age range (41–60 yrs) [23], was 47.5 years. Taking the two age-estimation results together, we considered that the age at death was middle-aged and less than 50 years (S2 Table). The individual’s stature was estimated to have been 153.0 cm. All of the anthropological data are available in summarized form in S3 Table.

**Fig 2:**
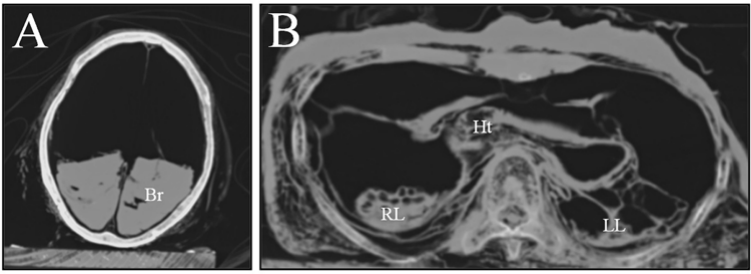
The CT images of this case. (A) for head; (B) for thorax. Most mummified organs were displaced to the dorsal side of body cavities. Their morphologies were seriously deformed. Br, brain; Ht, heart; RL, right lung; LL, left lung.

**Fig 3:**
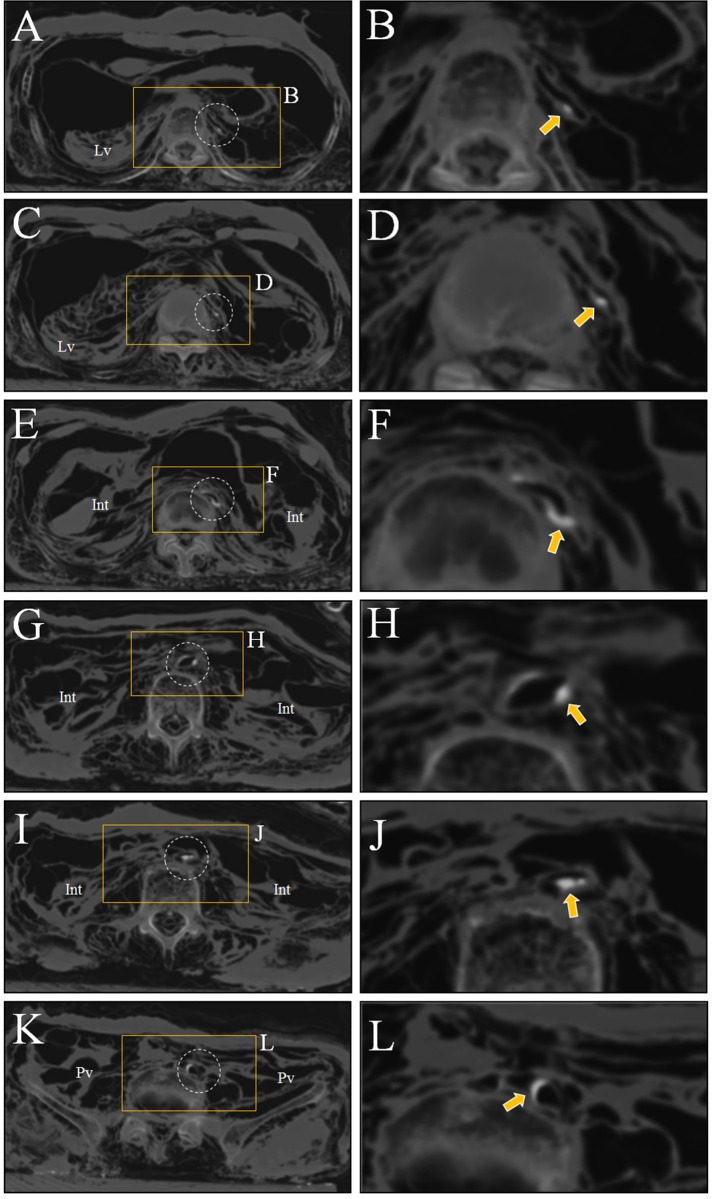
The CT images showing calcifications in aorta wall. (A) to (D) Upper abdomen. Calcifications (pointed by yellow arrows) are seen in the wall of abdominal aorta (dotted circles). (B) and (D) are magnified images of (A) and (C), respectively. Lv, liver. (E) to (H) The axial view through umbilicus. Calcifications are still identified in aorta walls. Int, intestines. (F) and (H) are magnified image of (E) and (G), respectively. (I) to (L) The axial view of pelvic region. Aortic calcification could be also observed. (J) and (L) are magnified images of (I) and (K), respectively. Pv, pelvis.

Computed tomography (CT) images showed patterns similar to those seen in other Korean mummies. The mummified organs in the thoracic, abdominal and pelvic cavities were displaced to the dorsal side, possibly due to the long-term effect of gravitational force. Several of these organs, such as the lungs, liver, intestines, and heart, were clearly visible even if their shapes had been seriously deformed, probably by dehydration and displacement (Fig. 2). The CT images also revealed many calcifications in the aortic wall, which were deemed likely to have protruded into the vascular lumen (Fig. 3). These calcifications were considered to be probable pathognomonic signs of atherosclerosis, though that hypothesis could not be confirmed. In the case of smaller vessels, little evidence of microvascular angiopathy on the CT images could be found.

**Fig 4:**
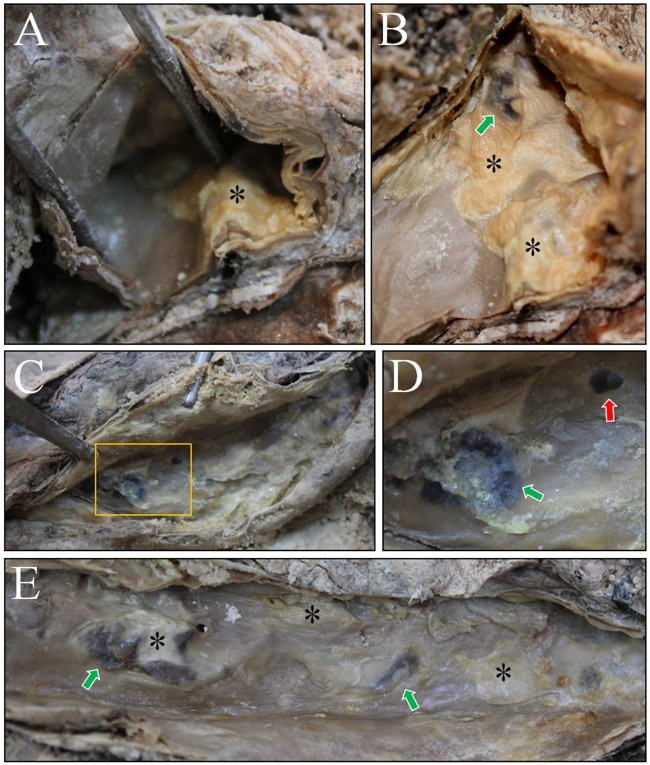
Dissection on the thoracic and abdominal cavity. The luminal surface of aorta was examined. (A) and (B) Atheromatous plaques (asterisks) exhibiting intimal thickness could be observed in aortic arch. Green arrow indicates possible hemorrhage. (C) and (D) Dissection of abdominal aorta. Atheromatous plaques could be identified (rectangle in C). (D) is the magnified image. Note the atheromatous plaque (asterisk) also showing hemorrhages (green arrows). Red arrows indicate ostia of the vessels. (E) Atheromatous plaques (asterisks) in the abdominal aorta. Green arrow indicates the hemorrhage.

Dissection subsequently was performed to determine the nature of the aortic-wall calcifications. First, upon dissection of the thoracic aorta, atheromatous plaques showing intimal thickening were noted (Figs. 4A and 4B). And hemorrhages in the atheroma were also identified (Fig. 4B). In an examination of the abdominal aorta, pale-yellow atheromatous plaques were found in the intima. Hemorrhages and rough ulcerated plaques also were evident (Figs. 4C-4E).

**Fig 5:**
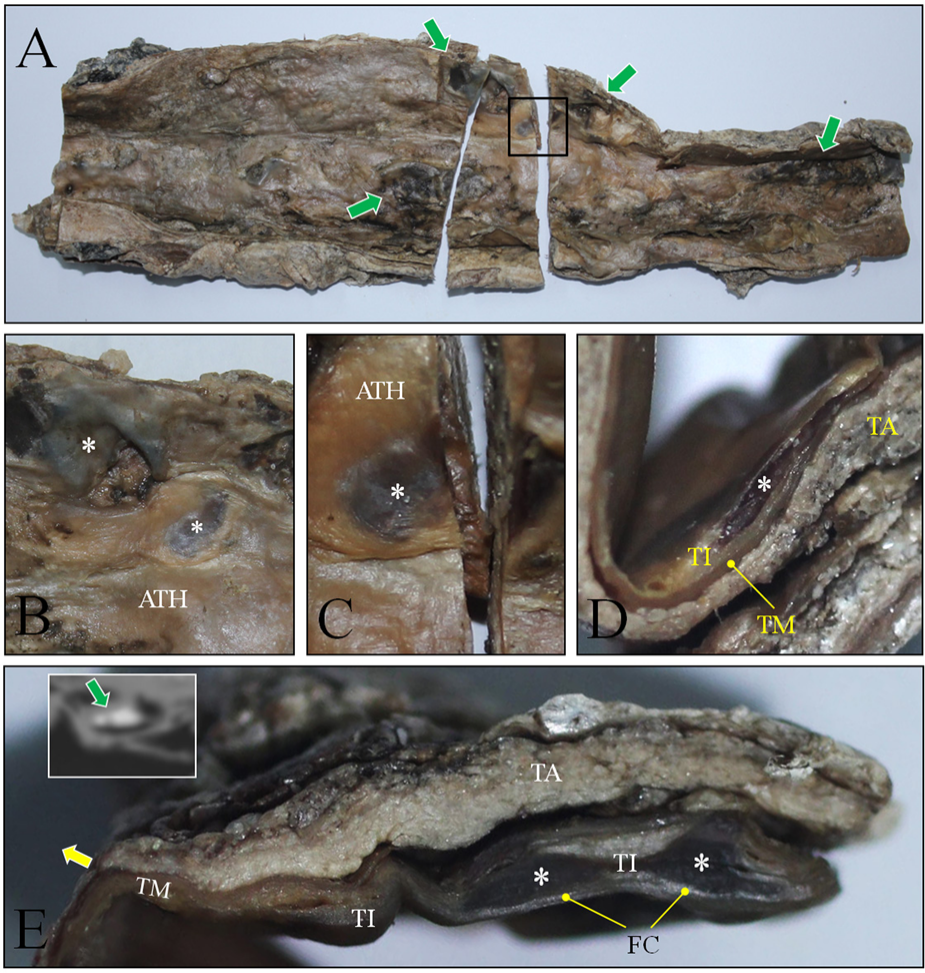
Cross-section of abdominal aorta. (A) Longitudinal section was done on the anterior wall to expose the inside of aorta. Many atheromatous plaques (indicated by green arrows) were seen on the aorta wall. (B) and (C) Magnified image of fully developed atheromatous plaque (ATH) Asterisks indicate the area where necrotic center is present beneath the fibrous cap. (D) Cross-section view of atheroma in (C). Asterisk indicate the same necrotic center seen in (C). TI, TM and TA are tunica intima, tunica media and tunica adventitia, respectively. (E) Magnified image of another atheroma. Asterisks indicate necrotic centers beneath the fibrous cap (FC). Inset is the CT image of aorta. The level of CT image is similar to that of cross-section (E). Green arrow indicates calcifications in aorta wall. They correspond to the necrotic centers seen in cross-section (E).

Next, we examined cross-sections of a vessel indicating intimal thickening. Atheromatous plaques on the endothelial surfaces of the abdominal aortic wall were evident (Fig. 5A). In the plaques, possible necrotic centers covered by the fibrous cap were apparent (Figs. 5B and 5C). The necrotic center in the tunica intima was confirmed by cross-sectioning (Fig. 5D). Atheromatous plaque with a necrotic core covered by the fibrous cap also was identified in the intimal thickening of another aortic cross-section. We speculated that such necrotic areas might also be evident on the corresponding CT-image calcifications, which proved to be the case (Fig. 5E).

**Fig 6:**
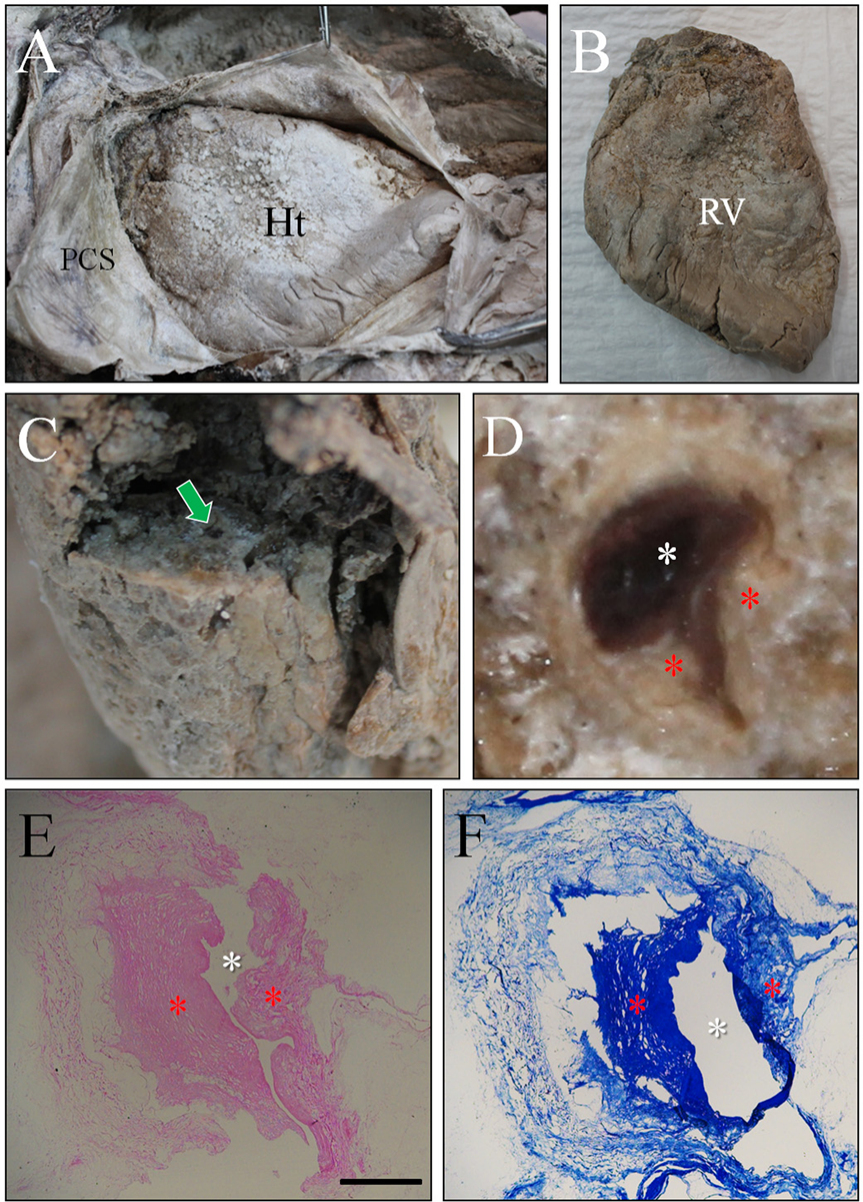
Gross appearance of heart and its coronary arterial sclerosis with histological findings. (A) Dissection of heart. PCS, pericardial sac; Ht, heart. (B) Removal of heart. RV, right ventricle. (C) Dissection of left anterior descending artery (indicated by green arrow). (D) Magnified image of cross-section of the artery. White asterisk indicate the blood clot filled in the coronary artery. Yellow asterisks indicate the intimal thickening. (E) is cross-section of the artery stained by H and E. (F) is the histology of the same artery stained by Masson’s trichrome staining. Scale bar = 25 μm.

Finally, we endeavored to determine whether the mummy had suffered from any coronary artery disease. The mummified heart was well-preserved within the pericardial sac (Figs. 6A and 6B). Dissection of its anterior side revealed the LAD artery (Fig. 6C), a cross-section of which clearly showed intimal thickening in the lumen (Fig. 6D). Under hematoxylin/eosin and Masson’s trichrome staining, intimal thickening of the coronary artery also was obvious (Figs. 6E and 6F). In our macroscopic and histological studies on the heart, however, we could not find any evidence of myocardial fibrosis/scarring.

## Discussion

Embalmers in ancient civilizations customarily removed the internal organs from bodies in the course of well-developed and often ritualistic mummification procedures (*e*.*g*., as in ancient Egypt). Whereas these procedures efficiently inhibited proliferation of microorganisms in the bowel, thereby facilitating successful mummification, they denied later researchers any mummified organs for examination. Indeed, the precise diagnosis of mummy pathologies or causes of death is difficult in general.

Even so, relatively exceptional cases provide researchers with invaluable internal organs still remnant in body cavities [24–28]. Over the past several years, researchers in Korea have also been fortunate to find Joseon mummies exhibiting excellent preservation statuses even after considerably long burial periods. Most of the internal organs in these Korean mummies were evident and even intact, as Joseon society never removed them for the sake of any preservation imperative. Although the precise mechanism of mummification in Korea is as yet unknown, studying these mummies can reveal many and specific details on the health (and disease) status of the Joseon people [11, 20, 21, 29–43].

Like other mummy researchers, when blessed with the rare opportunity to study a well-preserved mummy, Korean scholars have preferred to examine them first by CT, as this method can reveal the preservation statuses of internal organs while avoiding or minimizing damage [11, 29, 32, 36, 37, 41–43]. In the current, 17^th^ century Korean mummy case, we also performed a preliminary CT scan, during which we discovered multiple calcifications in the aortic walls. Such vascular calcifications on CT images have been regarded as top pathognomonic signs of ancient atherosclerosis [3–8].

Judging from our experience of the past several years however, correct interpretation of mummy CT findings is problematic. A key factor is that mummified organs typically show morphological deformation caused by post-burial processes [11, 44–47]. Accumulation of as much dissection data as possible, therefore, becomes crucial to the confirmative diagnosis of atherosclerosis. Even though vascular calcification is certainly among the strongest indicators of ancient atherosclerosis, definitive diagnosis still requires supplementary indicative signs, two of which are dissection and histological findings. Fortunately enough for us, and thanks to the family’s gracious consent, we were able to perform a dissection in the present case.

The key pathological characteristics of *modern* atherosclerosis are known to be intimal thickening and lipid accumulation in the aortic wall. Atheromatous plaque consists of a raised necrotic core involving the intima, which is further covered by a firm white fibrous cap. Even though they are distributed initially in focal and sparse patterns, these lesions become increasingly numerous and diffuse as the disease progresses [1].

Notably, every unique pathological sign of atherosclerosis noted above was identified in our dissection. Atheromatous plaques were found in all of the mummified aortic samples we examined. Typical signs of atherosclerosis, such as yellow fatty streaks, hemorrhage and ulceration, also were evident on the intimal surfaces. Cross-sections of the vessels indicated atheromatous plaque and its necrotic core in the intima, but not in the tunica media or adventitia of the mummy aorta. The suspected atherosclerotic origin of the calcifications was supported by our dissection of the aorta and examination of the vascular intima. Utilizing CT and dissection techniques together in our study, we concluded that the Korean mummy probably suffered from aortic and coronary atherosclerosis in her lifetime.

However, we must also consider the fact that atherosclerosis in younger females is known to be rare in general. Actually, women typically develop atherosclerosis-based coronary artery disease about 10 years later than men, due to possible hormonal protection [48]. Considering the estimated age at death of the current mummy, less than 50 years, seriously calcified and ulcerated atherosclerosis does seem very exceptional.

Nevertheless, it is also true that atherosclerosis is (only) *relatively rare* among younger women, not completely absent. For example, in the Egyptian mummy Lady Rai (1570–1530 BCE), who likely died while in her 30s, calcified atherosclerotic plaques were clearly observable in CT images of the abdominal aorta, indicating atherosclerosis as a probable cause of death [5]. In fact, given that significant atherosclerosis is not-so-rarely associated with younger female patients who suffer from hypertension, diabetes, hypercholesterolemia and low-normal thyroid function [49–54], we could not rule out ancient atherosclerosis as the cause of death in the present case, based only on the individual’s relative youth at the time of death.

Recent studies worldwide have established that atherosclerosis was not a rare affliction of ancient peoples [6, 8]. Those studies have shown that even though the environmental factors predisposing people to atherosclerosis have grown stronger over the centuries, the disease is not characteristic of modern society *per se*. Likewise, our research also shows that pre-modern peoples in Korea, notwithstanding the lesser environmental risk factors relative to those faced by their modern-day descendants, must have suffered from atherosclerosis. This current first-ever report of probable ancient atherosclerosis in an East Asian country establishes that East Asian cultures also, not just Western ones, were susceptible to the disease even prior to their modernization.

## Conclusion

In the light of our experiences, the value of CT analysis as an initial-diagnostic tool for the study of ancient atherosclerosis should not be underestimated. Especially where no definitive diagnosis can be made via invasive techniques like dissection, CT could be the one and only option remaining to researchers seeking to diagnose atherosclerosis non-invasively. The utility of CT imaging to the preliminary diagnosis of ancient atherosclerosis could be clearly elucidated by the current report.

## Supporting Information

S1 Table Sex estimation by the morphology of hip bone. (DOC)

S2 Table Age estimation based on osteological evidences. (DOC)

S3 Table Anthropometric Data. (DOC)
