# Supplementary material for: Anatomical Confirmation of Computed Tomography-Based Diagnosis of the Atherosclerosis Discovered in 17th Century Korean Mummy
Source: PLoS One. 2015 Mar 27;10(3):e0119474. doi: 10.1371/journal.pone.0119474 (PMC4376940; doi:10.1371/journal.pone.0119474)
Supplement: S1 Table — (DOC) [file pone.0119474.s001.doc]

Table S1. Sex estimation by the morphology of hip bone

| Name | Pre-auricular sulcus | Subpubic  angle | Ischiopubic ramus | Subpubic concavity | Ventral arc | Decision |
| --- | --- | --- | --- | --- | --- | --- |
| Mungyeong | present | wide | sharp | present | present | Female |
