# Supplementary material for: Anatomical Confirmation of Computed Tomography-Based Diagnosis of the Atherosclerosis Discovered in 17th Century Korean Mummy
Source: PLoS One. 2015 Mar 27;10(3):e0119474. doi: 10.1371/journal.pone.0119474 (PMC4376940; doi:10.1371/journal.pone.0119474)
Supplement: S2 Table — (DOC) [file pone.0119474.s002.doc]

S2 Table. Age estimation based on osteological evidences

| Method | Description and Age Estimation | | Age Estimation |
| --- | --- | --- | --- |
| Auricular Surface  (Lovejoy et al., 1985) | Transverse organization | No billows, vague striae | Phase V  (40-44 yrs) |
|  | Porosity | Micro, maybe macro |  |
|  | Granularity | Transition from granular to dense |  |
|  | Retroauricular activity | Slight to moderate |  |
|  | Apical activity | slight |  |
| Pubic  Symphyseal Surface  (Brooks and Suchey, 1990) | Symphyseal face | No ridge with fine granularity | Phase IV  (38.2 ± 10.9 yrs) |
|  | Ventral border | Distinct outline and no erosion but hiatus exist |  |
|  | Dorsal border | Distinct outline and no lipping with osteophyte |  |
| Right Maxillary Premolar Examined by Method of Lamendin et al. (1992) | Root height | 12.03mm | 47.5 yrs^a^ |
|  | Periodontosis height | 2.28mm |  |
|  | Transparency height | 5.32mm |  |

^a^Calculated as = (0.18 x P) + (0.42 x T) + 25.53; P = (Periodontosis Height/Root Height)x100; T = (Transparency Height/Root Height) x 100, following Lamendin et al. (1992).
