# Supplementary material for: Anatomical Confirmation of Computed Tomography-Based Diagnosis of the Atherosclerosis Discovered in 17th Century Korean Mummy
Source: PLoS One. 2015 Mar 27;10(3):e0119474. doi: 10.1371/journal.pone.0119474 (PMC4376940; doi:10.1371/journal.pone.0119474)
Supplement: S3 Table — (DOC) [file pone.0119474.s003.doc]

S3 Table. Anthropometric Data (cm)

| Height | 150.0 |
| --- | --- |
| Anterior trunk height | 52.0 |
| Thoracic Height | 18.7 |
| Abdominal Height | 33.3 |
| Acrominale Height (R/L) | 130.8 |
| Radiale Height (R/L) | 103.7 |
| Stylion Height (R/L) | 84.5 |
| Dactylion Height (R/L) | 67.4 |
| Ilioscristale Height (R/L) | 93.2 |
| Trochanterion Height (R/L) | 84.3 |
| Tibiale Height (R/L) | 39.7 |
| Biacromial Breadth | 29.2 |
| Bicristal Breadth | 29.4 |
| Upper Arm Length (R/L) | Lt. 28.5 / Rt. 30.9 |
| Lower Arm Length (R/L) | Lt. 21.0 / Rt. 21.2 |
| Total Arm Length (R/L) | Lt. 49.5 / Rt. 52.1 |
| Total Upper Extremity Length (R/L) | Lt. 68.7 / Rt. 70.8 |
| Hand Length (R/L) | Lt. 19.2 / Rt. 18.7 |
| Hand Breadth (R/L) | Lt. 9.0 / Rt. 8.8 |
| Thigh Height (R/L) | Lt. 40.3 / Rt. 39.0 |
| Leg Height (R/L) | Lt. 29.6 / Rt. 29.6 |
| Width of the Head | 14.2 |
| Bitragion Diameter | 14.8 |
| Forehead Height | 15.4 |
| Special Height of the Head | 15.1 |
| Height of the Head and Nose | 18.4 |
| Height of the Head and Face | 23.6 |
| Length of Head | 17.5 |
| Circumference of the Head | 53.7 |
| Distance Vertex to Tragion (R/L) | Lt. 14.2 / Rt.14.2 |
| Width of the Face | 12.9 |
| Width of the Mandible | 14.0 |
| Height of the face | 11.2 |
| Height of the Upper face | 5.0 |
| Height of the Lower face | 6.6 |
| Lower Half of the Craniofacial Height | 10.7 |
| Supraorbital Arc | 29.9 |
| Maxillary Arc | 28.3 |
| Mandibular Arc | 31.2 |
| Intercanthal Width | 3.4 |
| Length of the Eye Fissure (R/L) | Lt. 2.9 / Rt. 3.3 |
| Orbito-Tragion Distace (R/L) | Lt.8.1 / Rt. 8.6 |
| Orbito-Gonial Distace (R/L) | Lt. 9.2 / Rt. 9.2 |
| Orbito-Glabella Distace (R/L) | Lt. 5.7 / Rt. 5.7 |
| Anatomical Width of the Nose | 3.6 |
